# Zebrafish Transgenic Line *huORFZ* Is an Effective Living Bioindicator for Detecting Environmental Toxicants

**DOI:** 10.1371/journal.pone.0090160

**Published:** 2014-03-03

**Authors:** Hung-Chieh Lee, Po-Nien Lu, Hui-Lan Huang, Chien Chu, Hong-Ping Li, Huai-Jen Tsai

**Affiliations:** 1 Institute of Molecular and Cellular Biology, National Taiwan University, Taipei, Taiwan; 2 Liver Disease Prevention & Treatment Research Foundation, Taipei, Taiwan; 3 Taiwan Agricultural Chemicals and Toxic Substances Research Institute Council of Agriculture, Executive Yuan, Taichung, Taiwan; 4 Department of Soil and Environmental Sciences, National Chung Hsing University, Taichung, Taiwan; National University of Singapore, Singapore

## Abstract

Reliable animal models are invaluable for monitoring the extent of pollution in the aquatic environment. In this study, we demonstrated the potential of *huORFZ*, a novel transgenic zebrafish line that harbors a human upstream open reading frame of the *chop* gene fused with GFP reporter, as an animal model for monitoring environmental pollutants and stress-related cellular processes. When *huORFZ* embryos were kept under normal condition, no leaked GFP signal could be detected. When treated with hazardous chemicals, including heavy metals and endocrine-disrupting chemicals near their sublethal concentrations (LC50), *huORFZ* embryos exhibited different tissue-specific GFP expression patterns. For further analysis, copper (Cu^2+^), cadmium (Cd^2+^) and Chlorpyrifos were applied. Cu^2+^ triggered GFP responses in skin and muscle, whereas Cd^2+^ treatment triggered GFP responses in skin, olfactory epithelium and pronephric ducts. Moreover, fluorescence intensity, as exhibited by *huORFZ* embryos, was dose-dependent. After surviving treated embryos were returned to normal condition, survival rates, as well as TUNEL signals, returned to pretreatment levels with no significant morphological defects observed. Such results indicated the reversibility of treatment conditions used in this study, as long as embryos survived such conditions. Notably, GFP signals decreased along with recovery, suggesting that GFP signaling of *huORFZ* embryos likely reflected the overall physiological condition of the individual. To examine the performance of the *huORFZ* line under real-world conditions, we placed *huORFZ* embryos in different river water samples. We found that the *huORFZ* embryos correctly detected the presence of various kinds of pollutants. Based on these findings, we concluded that such uORF*^chop^*-based system can be integrated into a first-line water alarm system monitoring the discharge of hazardous pollutants.

## Introduction

Among the threats endangering aquatic environments, the discharge of industrial and domestic wastewater has the most significant impacts on freshwater ecosystems, as well as agricultural production and human health [1]–[4]. Thus, constant monitoring is essential to ensure timely response whenever damaging waste discharge events happen.

Chemical analysis is typically used to detect traces of known toxins in aquatic environments. However, this technique relies heavily on pre-established standards governing which chemicals and which concentrations are considered dangerous; therefore, it cannot be used to detect the existence of unexpected hazardous chemicals. In contrast, a biomonitoring system may reflect the subtle cellular and physiological changes occurring in living organisms when challenged by a variety of environmental pollutants.

Fish have been considered an ideal biomonitoring organism based on their biodiversity, population and health status. Using fish as an organism for *in vivo* toxicity assays has previously been proposed [5]. Normal conditions, such as growth, survival rates and egg hatchability, can be used as monitoring parameters. Quantifying the activity of enzymatic defenses in fish is also a common approach to assess water quality. However, the interpretation of the data obtained from these methods is limited by the fact that multiple physiological, genetic, and metabolic factors may simultaneously affect these multifunctional enzymes [6]–[12]. For example, mixed-function oxygenase (MFO), or mono-oxygenase, are important components of many metabolic systems and have been validated in a large number of field studies worldwide. However, the enzyme activities of MFO components, which contain cytochrome P450, cytochrome b5 and NADPH-cytochrome C reductase, must be measured individually to obtain the biomonitoring index. Moreover, tissue samples must be handled with great care to guard against denaturation and/or proteolysis. To overcome these limitations, transgenic fish lines have been developed by using native gene promoters, including the *cyp1a1* promoter, which is induced by polycyclic aromatic hydrocarbons [13], [14], or the *heat-shock* promoter, which is induced by heat and other stressors [15]. However, since these promoters only respond to specific forms of stress, their advantages over traditional chemical analysis are not particularly significant. Apart from this consideration, it is also true that a given stress with little harm to the animal may still induce the expression of a reporter gene controlled by the *heat-shock* promoter [15]. Under these circumstances, the reporter activity would have little relationship to the actual physiological stresses. Hence, for an animal model to be a practical biomonitor, it must 1) respond to a wide range of pollutants with accuracy and sensitivity and 2) dynamically trace physiological stresses.

In recognition of these objectives, we took advantage of a zebrafish transgenic line *huORFZ*, which harbors a GFP transgene regulated by the upstream open reading frame (uORF) fragment of human *DNA-damage-inducible transcript 3* (*ddit3*, previously named *chop*) cDNA [16]. Recent studies have shown that uORF-based translational regulation plays significant, if not primary, roles in the production of CHOP protein [17]-[19], while the *chop* gene is one of the most commonly used biomarkers for endoplasmic reticulum (ER) stress [20], [21]. We found that embryos derived from the *huORFZ* line only display fluorescent signals upon encountering stresses, with no detectable leakage under normal condition. Thus, *huORFZ* embryos can give a faithful account of cellular stresses. Using *in vivo* imaging, we further demonstrated that this line could be used to detect various environmental contaminants, including heavy metals and endocrine-disrupting chemicals (EDCs). Depending upon the treatment time, the limits of detection (LODs) for several common pollutants examined in this study were equal to, or below, World Health Organization (WHO) drinking water standard [22]. Importantly, different stresses were found to cause different GFP expression patterns in a dose-dependent manner. Moreover, after surviving treated embryos were returned to normal condition, survival rates, as well as TUNEL signals, returned to pretreatment levels with no significant morphological defects observed. Such results indicated the reversibility of treatment conditions used in this study, as long as embryos survived such conditions. Notably, GFP signals decreased along with recovery, suggesting that GFP signaling of *huORFZ* embryos likely reflected the overall physiological condition of the individual. Therefore, since time-consuming and complex analysis in various physiological conditions may not be necessary, the use of the *huORFZ* embryos holds considerable promise as a novel fluorescent biomonitoring method.

## Materials and Methods

### Ethics Statement

The animal protocol, which was strictly followed in this study, was reviewed and approved by the IACUC, National Taiwan University, Taiwan, with approval number NTU-102-EL-19.

### Animal husbandry

All wild-type zebrafish (*Danio rerio*) were AB/TU strains, and transgenic lines of zebrafish *Tg(-2.9krt18:RFP)* [23] and *huORFZ* [16] were crossed into the AB/TU background. All fish were maintained at a temperature of 28.5°C with a photoperiod of 14 hr light:10 hr dark. All fish were bred according to guidelines outlined in *The Zebrafish Book* [24]. Embryos were raised in embryo medium (140 mM NaCl, 5.4 mM KCl, 0.25 mM Na_2_HPO_4_, 0.44 mM KH_2_PO_4_, 1.3 mM CaCl_2_, 1.0 mM MgSO_4_, and 4.2 mM NaHCO_3_ at pH 7.2) until 24 hr post-fertilization (hpf), followed by incubation in embryo medium containing 0.003% 1-phenyl-2-thiourea (Sigma) to prevent pigment formation.

### Chemicals

Lithium chloride (Sigma), copper sulfate pentahydrate (Sigma), nickel (II) sulfate hexahydrate (Sigma), zinc sulfate (Merck), aluminum chloride hexahydrate (Sigma), cobalt chloride hexahydrate (Sigma), lead chloride (Sigma) and acrylamide (Sigma) were used for the toxicity tests. Cadmium chloride (Merck), arsenic trioxide (Merck), atrazine (EQ Laboratories), chlorpyrifos (EQ Laboratories), carbofuran (Chem Service), dimethoate (Chem Service), glyphosate (EQ Laboratories), and methoxychlor (Fluka) were provided by Taiwan Agricultural Chemicals and the Toxic Substances Research Institute, Council of Agriculture, Taiwan. Heavy metals, acrylamide, dimethoate and glyphosate were dissolved in sterile distilled water to make stock solutions, whereas other chemicals were either dissolved or diluted with dimethyl sulfoxide (DMSO) to the desired concentration as stock solutions.

### Stress treatment

All embryos were raised in 10-cm Petri dishes and incubated at 28.5°C. All of the toxicity tests began at 72 hpf. Treatments were performed in 3-cm Petri dishes containing 20 embryos. The embryos were first washed once with 3 mL of distilled water. After washing, the liquid was removed, leaving the least amount possible in the dish. Then 3mL of working solution were added to each dish. Control embryos were treated with the equivalent amount of sterile distilled water or DMSO-containing water. The embryos were then returned to the incubator set to standard zebrafish embryo culture condition (28°C). Unless otherwise stated, each experiment consisted of 60 healthy embryos divided into three groups (n = 20 embryos per group) for each treatment condition. Each experimental design was repeated three times independently, and the results were pooled to calculate the percentage. Lethal concentrations for 10%, 50% and 90% mortality were calculated from a linear regression of log probit transformations of the dose response data [25]. The final chemical concentrations of heavy metals and EDCs used in this study are listed in Table S1. In the survival rate experiment, five independent repetitions of 20 embryos for each treatment condition were performed. Data presented as mean±SD in the manuscript were the averages of these five independent experiments, which were carried out using different clutches of embryos on different dates. For the recovery experiment, one dish of *huORFZ* embryos remained under stress throughout the experiment in each repetition for each kind of stress, while the other dish was washed twice with sterile distilled water and returned to normal condition at the end of each specific treatment.

Chlorpyrifos was chosen as a representative of chronic toxicity for testing in *huORFZ* larvae. Embryos at 72 hpf were treated with chlorpyrifos at 86 nM, according to the values for drinking water quality, as specified in the WHO guidelines, and observed for three days. Solution was refreshed daily. For the semi-quantitative experiment, two independent repetitions of 20 embryos for each treatment condition were performed. Images of three to four randomly selected live embryos were taken from each treatment condition and each repetition.

### Western blotting

Zebrafish embryos were lysed by 1 x whole cell extract buffer (15 mM Tris-HCl pH 7.5, 250 mM sucrose, 2 mM EDTA, and 0.2 mM PMSF), and proteins were separated by 10% SDS-PAGE, followed by transfer to PolyScreen PVDF Hybridization Transfer Membrane (PerkinElmer). Subsequently, the membranes were probed with antiserum, including 1∶1000 diluted anti-GFP (Chemicon Millipore), 1∶500 diluted anti-GADD153 (mouse CHOP homologue) (Abcam ab11419) and 1∶5000 diluted anti-α-tubulin (Sigma). Membranes were washed with TBST solution (0.2 M Tris, 1.37 M NaCl and 0.1% Tween-20, pH 7.6) and probed with 1∶5000 diluted horseradish peroxidase-conjugated goat anti-mouse antibody (Santa Cruz Biotechnology). The bound antibody was detected by Western Lightning ECL Pro (PerkinElmer) and then exposed to X-ray film (Fujifilm).

### Immunohistochemistry

*huORFZ* larvae at 4-days post-fertilization (dpf) were fixed with 4% paraformaldehyde (PFA) overnight at 4°C and cryoprotected by 30% sucrose before sectioning at 20 µm horizontally on a cryostat. Slides were washed with phosphate-buffered saline (PBS) for 30 min. They were subsequently incubated in blocking solution (PBS, 2% Bovine serum albumin, 0.2% Triton X-100) for 30 min. The following primary antibodies were used: anti-GFP (Abcam; 1∶200) and mouse glutamine synthetase (GS) (clone GS-6; 1∶500). The immunohistochemical signals were detected with FITC-conjugated secondary antibody (Santa Cruz Biotechnology) for GFP and Cy3-conjugated secondary antibody (Millipore) for GS. Nuclei were counterstained with 4′,6-diamidino-2-phenylindole (DAPI) (Sigma).

### TUNEL staining

*huORFZ* larvae were fixed and cryoprotected following the same protocol described above. Whole mount TUNEL was carried out on Cu^2+^-treated embryos. Cd^2+^-treated embryos were coronally sliced, and TUNEL staining was performed following the manufacturer's protocol (In Situ Cell Death Detection Kit TMR red, Roche). Slides were washed three times with PBST for 5 min each, and the images were examined by confocal microscopy.

### Microscopy and imaging

Fluorescent images were captured by a fluorescent stereomicroscope (MZ FLIII, Leica) coupled with Nikon D3 digital camera or a confocal spectral microscope (LSM 780, Zeiss). Unless otherwise indicated, representative images used in this study represent more than 75% of the embryos in an experimental group.

### Semi-quantification analysis based on fluorescent images

The analysis was modified from the procedure described by Noche (2011) [26]. Briefly, green fluorescent images were taken under a Leica MZ FLIII fluorescent stereomicroscope with objective set to 4X. All images were lateral view with ISO 3200 and 4 seconds of exposure. Images were saved as 24-bit RGB TIFF files.

The images were then opened under ImageJ 1.47 [27]. “Split channel” function was then used to extract the green channel, while the blue and red channels were discarded. The outline of the one embryo to be analyzed was loosely selected using the “Polygon selections” tool. Only the area more anterior to the most posterior end of the yolk stalk was selected. This arbitrary selection rule was applied to address the size of embryos in relation to the width of view under 4× magnitude. The “Rolling Ball Background” filter (algorithm based on Sternberg, 1983 [28]) with the rolling ball radius set to 35 was then used to subtract the background. After background correction, under the Brightness/Contrast window, the “Minimum” value was arbitrarily set to 10 to further eliminate remaining background value. A previously selected area was then recalled using the “Restore selection” function, and the “Measure” function was used to obtain the “Integrated density” value for further analysis.

### River water samples

River samples collected from Station 1, 2 and 3, designated as Sample 1, 2 and 3, respectively, were purchased from CENPRO Technology Co., Ltd. These samples were collected by Water Quality Monitoring Stations of the Environmental Protection Administration of Taiwan. Sample 4 was collected by H.L. Huang. Details on the sampling station coordinate and data are listed in Table S2. All samples were refrigerated for one to three days before biological toxicity assessments were conducted using *huORFZ* embryos and chemical analyses. River water samples for chemical analysis were additionally treated with concentrated nitric acid to digest the heavy metals.

### Quantification of the concentrations of heavy metals

Heavy metal measurements were carried out using Inductively Coupled Plasma-Mass Spectroscopy (ICP-MS, Agilent 7500 Series). Arsenic was determined by inductively coupled plasma atomic emission spectroscopy (ICP-AES, JY 138 UL Trace, France) equipped with a hydride generator. A hot plate was used for wet digestion of solution. All glassware and plastic were cleaned by Trace Clean (Milestone). Sampling cans and bottles were rinsed with deionized water and soaked in 3% HNO_3_ for 24 hr. After acid bath, the bottles for storage of precipitation samples were rinsed twice, filled with 1% HNO_3_, and plugged. Other containers and instruments were rinsed twice with deionized water, dried, plugged and packed in two clean plastic bags with zip locks. The rings and filter supports from the filter packs were soaked in 1% HNO_3_ for 12 hr and rinsed properly with deionized water. Autosampler tubes and cups were also rinsed with deionized water, soaked in 1% HNO_3_ at least 12 hr and rinsed twice with deionized water before use. Nitric acid (Ultrapure Reagent, 69%) was supplied by J.T. Baker. The metal standard was supplied by Merck ICP Multi Element Standard Solution XVI. For medium solution detection, approximately 5 mL of sample were digested with 10 mL of HNO_3_ on a hot plate. The temperature of the hot plate was maintained at 190°C for 1.5 hr. After cooling, the sample was diluted to 25 mL with distilled water. Metal contents of the final solution were determined by ICP-MS, as well as ICP-AES. The water samples were filtrated on membrane filters of 0.45 mm pore size, followed by determination of Pb^2+^, Zn^2+^, Cd^2+^, Cu^2+^, Ni^2+^ and As^3+^ concentrations by ICP-MS. The ions were separated by mass-to-charge ratio (m/z) and measured by a channel electron multiplier (Pb^2+^: 208 m/z, Zn^2+^: 66 m/z, Cd^2+^: 111 m/z, Cu^2+^: 63 m/z, Ni^2+^: 60 m/z, As^3+^: 75 m/z). The instrumental operating conditions for ICP-MS were as follows: Radio frequency power: 1500 W; RF matching: 1.75 V; Sample depth: 9 mm; Sample skimmer cones: Ni; Peristaltic pump: 0.10 rps; Argon plasma flow rate: 15 L/min; Auxiliary: 0.32 L/min; Nebulizer: 0.87 L/min; Spray chamber temperature: 2°C; Integration time: 0.1 s. The instrumental operating conditions for ICP-AES were as follows: Plasma power: 1100 W; Argon flow rate: 12 L/min; Nebulizer flow: 0.4 L/min; Nebulizer P: 2.5–3.5 bar; Wavelength: As 193.696 nm. An ICP multi-element standard solution (100 mg/L), containing the analyzed elements, was used in the preparation of calibration stock solutions. Working standard calibration solutions were prepared daily by dilution of the stock solutions, containing 50, 20, 10, 5, and 1 µg/L heavy metal solutions. The correlation coefficient r^2^ obtained for all cases was 0.9995. The LODs were calculated as the concentrations of an element that gave the standard deviation of a series of ten consecutive measurements of blank solutions.

### Statistical analysis

Unless otherwise indicated, each experiment was repeated three times or more. All bar graphs are presented as mean values with error bar indicating ± SD. Student's *t*-test was used for statistical analyses. Significance was determined at *P*<0.05 (*), *P*<0.01 (**) and *P*<0.001 (***).

## Results

### The GFP expression patterns in *huORFZ* embryos are tissue-specific responses to various stresses

**Figure 1:**
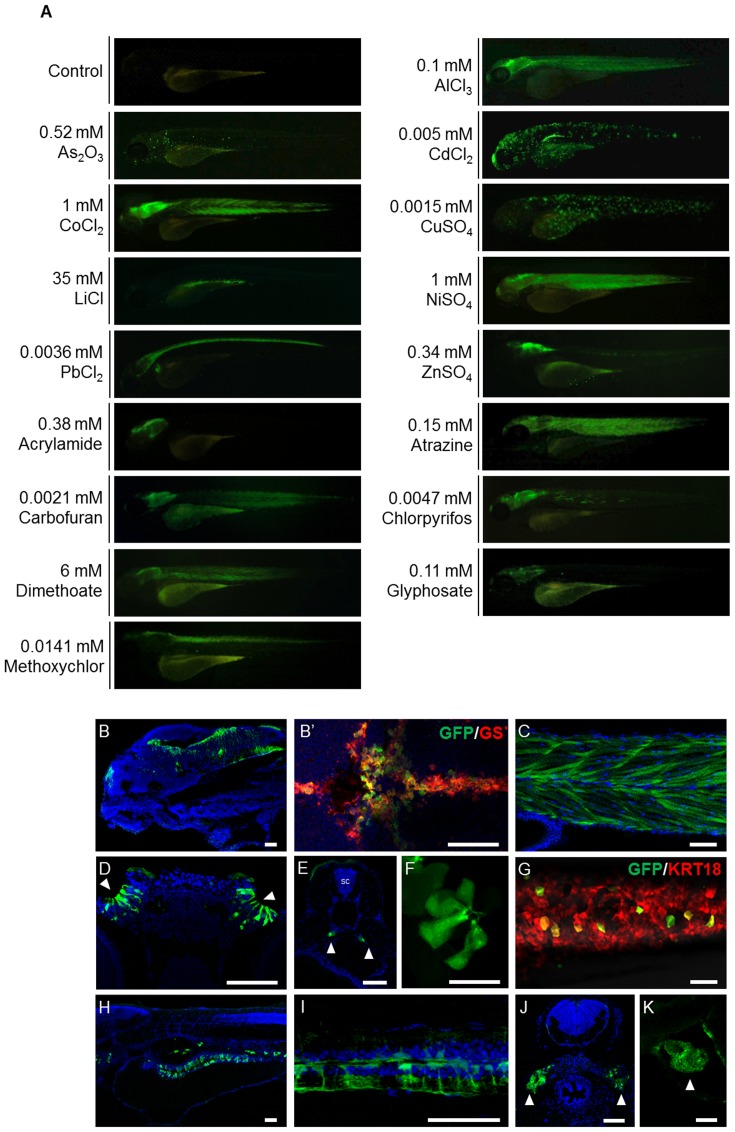
The GFP signals shown in *huORFZ* embryos are distinctly responsive to various stresses. (A). Various heavy metal-containing chemicals, including AlCl_3_, As_2_O_3_, CdCl_2_, CoCl_2_, CuSO_4_, LiCl, NiSO_4_, PbCl_2_ and ZnSO_4_, or different EDCs, including acrylamide, atrazine, carbofuran, chlorpyrifos, dimethoate, glyphosate and methoxychlor, were used individually to treat *huORFZ* embryos at 72 hpf. The concentrations of each heavy metal ion or EDC used were indicated on each panel. GFP expression patterns were observed at 96 hpf. The percentages of the representative patterns among the treatment groups are labeled on the images. (B-K). Selected GFP expression patterns were imaged in detail under confocal microscopy. For AlCl_3_-treated *huORFZ* embryos, GFP signals were observed in the brain (B) and muscle (C). Through immunostaining of antibody against GS, it was observed that only the glial cell lineages in the brain of embryos expressed GFP (B′; red labeling). For CdCl_2_-treated *huORFZ* embryos, GFP signals were detected in the olfactory epithelium (D; arrowhead), pronephric ducts (E; arrowhead), skin and lateral line system (F). For CuSO_4_-treatment, embryos obtained from crossing *huORFZ* to *Tg(-2.9krt18:RFP)* were used. When treated with CuSO_4_, GFP-expressing skin cells were always also *krt18*:RFP-expressing, indicating a keratinocyte lineage (G). For PbCl_2_-treated *huORFZ* embryos, the GFP-positive tissues included the spinal cord (I) and kidney (J; arrowhead). Interestingly, when either LiCl or Atrazine was used to treat the *huORFZ* embryos, GFP signals were observed in the intestine (H) and heart (K; arrowhead), respectively. (A, B, C, F – I, K) are lateral views with anterior to the left. (B′) is dorsal view with anterior to the left. (D, E, J) are transverse view with posterior to the top. SC: spinal cord. BR: brain. The scale bar in F is 20 µm; all other scale bars are 50 µm.

To evaluate whether *huORFZ* zebrafish embryos could be used to detect environmental pollutants *in vivo*, the heterozygous *huORFZ* embryos at 72 hpf were treated with common pollutants found in freshwater bodies, including heavy metals and EDCs. To determine the effective pollutant monitoring range, we first determined the lethal concentrations as 10%, 50% and 90% of each pollutant for 72 hpf embryos in a 24 hr treatment (Table S1). We then used a concentration equal to, or lower than, the LC_50_ of each pollutant for subsequent experiments. Control embryos in distilled water exhibited no GFP signals during the experimental period (Figure 1A, control). Intriguingly, among treated embryos, the GFP expression patterns varied according to the type of pollutants. Representative GFP expression patterns under each treatment condition are shown in Figures 1A and S1. Detailed distribution of GFP-responsive cells/tissues of each experimental group is shown in Figure 1B-K and summarized in Table 1. With heavy metal treatments, GFP signals were detected in the brain (Figure 1B) and muscle (Figure 1C) of the embryos treated with either 2.7 mg/L (0.1 mM) of Al^3+^ (from AlCl_3_) or 58.93 mg/L (1 mM) of Co^2+^ (from CoCl_2_). All of the GFP-positive cells in the brain reacted positively to the antibody against GS (Figure 1B′; red labeling), indicating a glial cell identity. When the embryos were incubated in 50 mg/L (0.67 mM) of As^3+^ (from As_2_O_3_), GFP signals were strongly expressed in the lateral line system. With 0.56 mg/L (0.005 mM) of Cd^2+^ (CdCl_2_) treatment, GFP signals were strongly expressed in the olfactory epithelium (Figure 1D), pronephric ducts (Figure 1E), skin and lateral line neuromasts (Figure 1F). When 58.69 mg/L (1 mM) of Ni^2+^ (from NiSO_4_) was used, GFP signals were detected in the olfactory epithelium, brain and muscle of the treated embryos. GFP was presented in a scattered form on the skin of embryos incubated with 0.1 mg/L (0.0015 mM) of Cu^2+^ (from CuSO_4_). The stress-induced GFP-expressing skin cells on the trunk colocalized with the expression of *keratin18* (*krt18*), as indicated by the expression of RFP in the embryos obtained from crossing *huORFZ* to *Tg(-2.9krt18:RFP)* (Figure 1G; red labeling), indicating the keratinocyte lineage of the GFP-expressing cells. GFP was also highly expressed in the intestine of embryos incubated with 242.9 mg/L (35 mM) of Li^+^ (from LiCl) (Figure 1H), and GFP responses were significant in the brain, spinal cord (Figure 1I) and kidney (Figure 1J) of embryos treated with 0.76 mg/L (0.0036 mM) of Pb^2+^ (from PbCl_2_). Finally, in embryos treated with 22.24 mg/L (0.34 mM) of Zn^2+^ (from ZnSO_4_), GFP was highly expressed in the brain, but only weakly detected in the spinal cord and yolk sac skin. The *huORFZ* embryos also responded to EDCs. For example, GFP was highly expressed in the brain of embryos treated with 26.98 mg/L (0.38 mM) of acrylamide, and while it was also strongly apparent in the brain and muscle of embryos treated with 32.35 mg/L (0.15 mM) of atrazine, GFP was only weakly apparent in the heart of these embryos (Figure 1K). GFP was also highly expressed in the brain and muscle of embryos treated with either 1.34 mg/L (0.0021 mM) carbofuran or 1375 mg/L (6 mM) dimethoate. Finally, GFP was strongly detected in the brain and some muscle fibers of embryos treated with 1.65 mg/L (0.0047 mM) chlorpyrifos. However, when treated with either 18.6 mg/L (0.11 mM) of glyphosate or 4.87 mg/L (0.0141 mM) of methoxychlor, GFP signals were only detected in the brain.

**Table 1:** GFP-expressing tissues in huORFZ embryos treated with different heavy metals and endocrine-disrupting chemicals (EDCs).

		Tissues of zebrafish embryo									
Treatments	Concentration (mg/L)	Olfactory epithelium	Brain	Spinal cord	Heart	Muscle	Skin	Lateral line system	Kidney	Pronephric duct	Intestine
**Heavy Metals**											
Al(III)	2.7	-	+*	**+**	**-**	**+**	**-**	**-**	**-**	**-**	**-**
As(III)	50	**-**	**-**	**-**	**-**	**-**	**-**	**+**	**-**	**-**	**-**
Cd(II)	0.56	+*	**-**	**-**	**-**	**-**	**+**	**+**	**-**	**+**	**-**
Co(II)	58.93	**-**	**+**	**+**	**-**	**+**	**-**	**-**	**-**	**-**	**-**
Cu(II)	0.1	**-**	**-**	**-**	**-**	**-**	+*	**+**	**-**	**-**	**-**
Li(I)	242.9	**-**	**-**	**-**	**+**	**-**	**-**	**-**	**-**	**-**	+*
Ni(II)	58.69	+*	**+**	**+**	**-**	**+**	**-**	**-**	**-**	**-**	**-**
Pb(II)	0.76	+	+*	**+**	**-**	**-**	**+**	**+**	**+**	**-**	**-**
Zn(II)	22.24	**-**	+*	**+**	**-**	**-**	**+**	**-**	**-**	**-**	**-**
**EDCs**											
Acrylamide	26.98	**-**	+*	**-**	**-**	**-**	**-**	**-**	**-**	**-**	**-**
Atrazine	32.35	**-**	+*	**-**	**-**	**+**	**-**	**-**	**-**	**-**	**-**
Carbofuran	1.34	**-**	+*	**-**	**-**	**+**	**-**	**-**	**-**	**-**	**-**
Chlorpyrifos	1.65	**+**	+*	**-**	**-**	**+**	**-**	**-**	**-**	**-**	**-**
Dimethoate	1375	**-**	**+**	**-**	**-**	**+**	**-**	**-**	**-**	**-**	**-**
Glyphosate	18.6	**-**	+*	**-**	**-**	**-**	**-**	**-**	**-**	**-**	**-**
Methoxychlor	4.87	**-**	+*	**+**	**-**	**-**	**-**	**-**	**-**	**-**	**-**

+:GFP signals are present; -: GFP signals are absent; *: Tissues that responded with highest sensitivity.

### The intensity of GFP signal in *huORFZ* embryos is correlated to the strength of stresses

**Figure 2:**
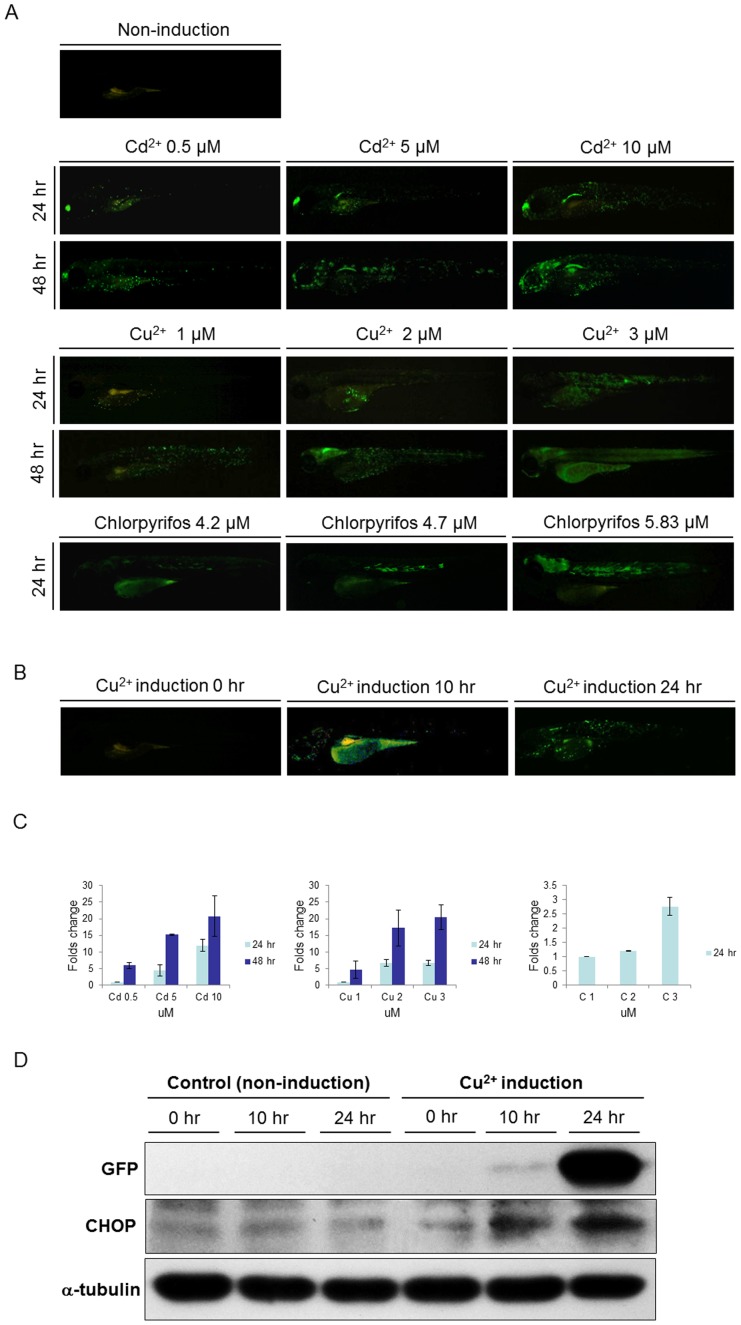
The intensity of GFP signal was positively correlated with the strength of stress and the expression of endogenous Ddit3. (A). At 72 hpf, *huORFZ* embryos were exposed to different concentrations of Cadmium (Cd^2+^), Copper (Cu^2+^), and chlorpyrifos, as indicated, and fluorescence signals were observed at 96 and 120 hpf. Mock group was treated with water containing DMSO which was added to the concentration representing the DMSO in the chlorpyrifos treatment group. As the chemical concentrations and incubation times increased, the GFP signals also increased. All images are lateral views with anterior to the left. (B). GFP expression patterns of *huORFZ* embryos after treatment with 0.1 mg/L (1.5 µM) of Cu^2+^ from 72 hpf for 10 hr and 24 hr, as indicated. All images are representative with the percentage among treatment groups labeled. All images are lateral views with anterior to the left. (C). The semi-quantification analysis based on fluorescent images. Both increased toxicant concentration and prolonged treatment time resulted in increased GFP signal intensity in *huORFZ* embryos. Note that the readings of each chemical treatment were normalized to the lowest dosage group of the same chemical. Thus, the signal values from different chemical treatments were not comparable. (D). The expression of endogenous Ddit3 in *huORFZ* embryos positively correlates to the signal strength of stress-induced GFP. Total cell lysates were prepared and analyzed by Western blot with specific antibodies to exogenous GFP and to the ER stress protein Ddit3. α-tubulin served as a loading control.

When *huORFZ* embryos were exposed to higher concentrations of toxic reagents, stronger fluorescence signals could be detected. The GFP signals also became detectable in more tissues (Figure 2A). For example, *huORFZ* embryos that were treated with 0.06 mg/L (0.5 mM) of Cd^2+^ for 24 hr displayed strong fluorescence signals in the olfactory epithelium, but only weak signals in the skin cells. However, when treated with 0.56 mg/L (5 mM) of Cd^2+^ for 24 hr, the *huORFZ* embryos displayed strong fluorescence signals in both olfactory epithelium and pronephric ducts. When the Cd^2+^ concentration was increased to 1.12 mg/L (10 mM), more skin cells began to exhibit GFP response. Also, when treatment time was extended to 48 hr, the GFP expression levels were correspondingly increased. Similarly, when the concentration of Cu^2+^ was increased from 0.06 mg/L (1 mM) to 0.19 mg/L (3 mM), more epithelial cells became GFP-positive. When the treatment time was extended to 48 hr, GFP signals became detectable in tissues other than epithelium. Specifically, the brain started to express GFP in the group treated with 0.13 mg/L (2 mM) of Cu^2+^ for 48 hr, while the muscle cells started to express GFP in the group treated with 0.19 mg/L (3 mM) of Cu^2+^ for 48 hr. A similar dose dependency was observed in chlorpyrifos-treated embryos. Again, as the concentration of chlorpyrifos was increased from 1.47 mg/L (4.2 mM) to 2.04 mg/L (5.83 mM), more tissues became GFP-positive. To better demonstrate the dose- and time-dependent effects of GFP signal strength in *huORFZ*, we conducted semi-quantitative analysis using fluorescent imaging. Three to four images from each treatment condition and each treatment time were taken and analyzed. The results confirmed our descriptional observation in that increased GFP signal follows increased dosage and treatment time (Figure 2C).

Furthermore, we found that the emergence of GFP signals over time (Figure 2B, C) correlated with the endogenous expression level of Ddit3, which is a known indicator of ER stress (Figure 2D). Such results suggest that the GFP response in *huORFZ* embryos may be seen as an indicator of ER stress. Thus, the induced GFP expression pattern in *huORFZ* embryos could be used to identify the type and dosage of the pollutant presented. In addition, based on the parameters suggested above, *huORFZ* embryos could potentially be employed to monitor the presence of poorly studied or previously unknown contaminants that induce ER stress.

### The GFP signals in *huORFZ* embryos indicate cells responding to acute and chronic toxic stress

**Figure 3:**
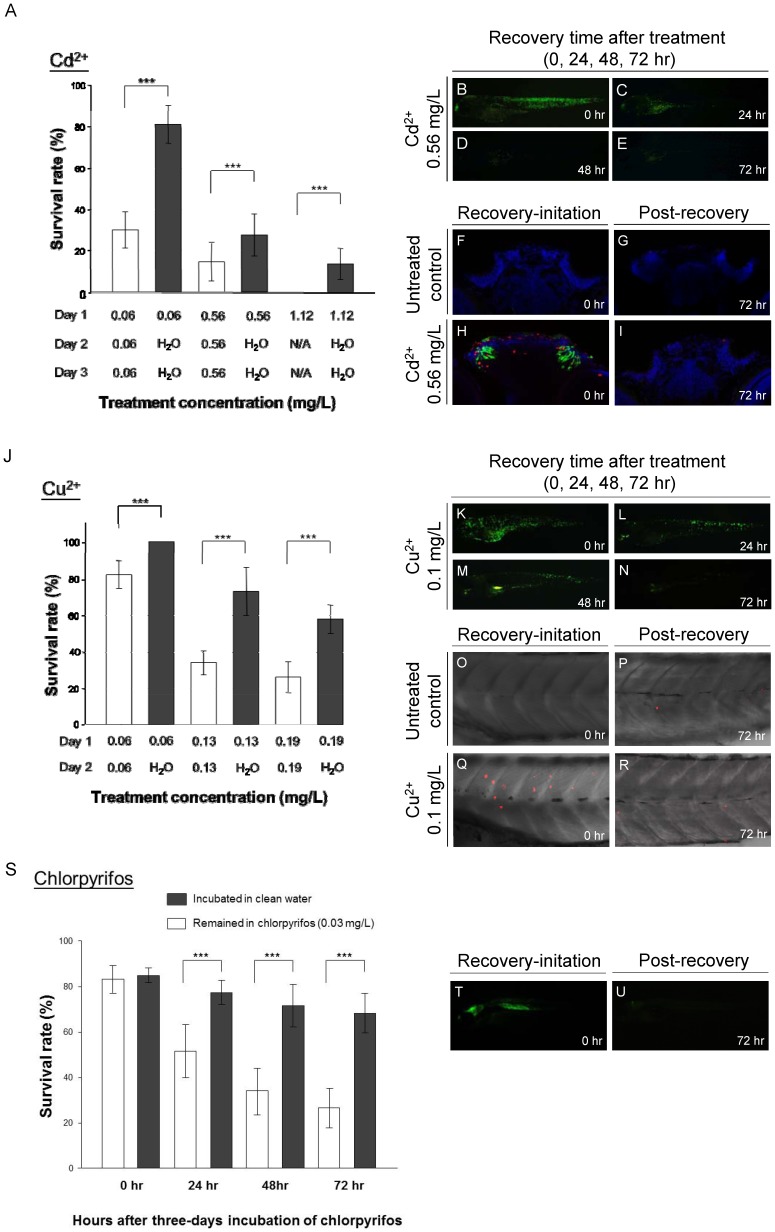
GFP expression in *huORFZ* as signals of cell under acute and chronic toxic stresses. At 72*huORFZ* embryos were treated with Cadmium (Cd^2+^; A-I) or Copper (Cu^2+^; J-R). (A). The survival rate of *huORFZ* embryos incubated in Cd^2+^ with different concentrations and treatment time. Open bars indicate *huORFZ* embryos that remained in the Cd^2+^ solution throughout the experiment; black bars indicate the embryos removed for recovery in clean water after 24 hr of Cd^2+^ treatment. N/A indicates that prolonged treatment was not conducted as a result of 100% lethality. (B-E). GFP signals immediately or 24, 48 and 72 hr after 24 hr of Cd^2+^ treatment. (F-I). TUNEL assay at the olfactory epithelium immediately or 72 hr after 24 hr of Cd^2+^ treatment. Red color represents TUNEL assay; Green color represents GFP signal; and Blue color represents DAPI staining. (J). The survival rates of *huORFZ* embryos incubated in Cu^2+^ with different concentrations and treatment times. The experimental strategy was the same as A-I. (K-N). GFP signals immediately or 24, 48 and 72 hr after 24 hr of Cu^2+^ treatment. (O-R). TUNEL assay at the skin immediately or 72 hr after 24 hr of Cu^2+^ treatment. (S-U). For the chronic toxicity test, 72 hpf *huORFZ* embryos were treated with chlorpyrifos with a concentration (86 nM) below WHO guidelines. (S). The survival rates of the embryos treated with chlorpyrifos for zero to three days. (T, U). GFP signals immediately or 72 hr after three days of chlorpyrifos treatment. (F-I). are transverse section with dorsal to the top. All other images are lateral views with anterior to the left. For each treatment (each bar in A, J and S), n = 100 embryos evenly distributed in five repetitions.

To confirm the correlation between the fluorescence performance of *huORFZ* embryos and their physiological responses to acute and chronic toxic challenge, embryos at 72 hpf were first incubated in solutions containing either Cd^2+^ (Figure 3A-I) or Cu^2+^ (Figure 3J-R) for at least 24 hr. Afterwards, a portion of the GFP-positive embryos were kept in the toxic reagents, while others were transferred to clean distilled water for recovery. The mortality of such recovering embryos was significantly lower than that of embryos left under continuous toxic challenge (Figure 3A, 3J). Additionally, the GFP signals decreased gradually after the GFP-expressing embryos were removed to clean distilled water, and GFP fluorescence could hardly be detected at 72 hr post-treatment of either Cd^2+^ (Figure 3B-E) or Cu^2+^ (Figure 3K-N). Apoptosis was next examined using the TUNEL assay following Cd^2+^ (Figure 3F-I) and Cu^2+^ treatments (Figure 3O-R). In the treatment group exposed over a period of 24 hr with an increased concentration of Cd^2+^ (0.56 mg/L, 5 µM), apoptosis was primarily restricted to the olfactory epithelium, as determined by transverse section through the forebrain (Figure 3H). In contrast, TUNEL signals were undetectable in the olfactory organ of larvae from the control groups (Figure 3F, 3G) and in larvae recovering 72 hr after Cd^2+^ exposure (Figure 3I). Similarly, in the treatment group exposed over a period of 24 hr with a concentration of 0.1 mg/L (1.5 µM) of Cu^2+^, TUNEL signals increased in skin cells when compared with control group (Figure 3Q *vs.* 3O). However, larvae recovering 72 hr after Cu^2+^ exposure showed few TUNEL signals (Figure 3R), and no significant difference was observed when compared with untreated larvae (Figure 3P), suggesting the abatement of stress-induced apoptosis as long as larvae were not continuously exposed to the metal contaminant and allowed to recover. Therefore, we concluded that the GFP expression of *huORFZ* embryos is likely a distress signal of cells responding to an external contaminant.

To examine the utility of *huORFZ* embryos as an organismal tool for monitoring chronic environmental stresses, embryos at 72 hpf were treated with 0.03 mg/L (86 nM) of chlorpyrifos for at least 72 hr. Then, a portion of the GFP-positive embryos were kept in the toxic reagent, while others were transferred to clean water to recover. The mortality of such recovering embryos was significantly lower than that of the group challenged by chronic toxic stress (Figure 3S). Additionally, the GFP signals decreased gradually after the GFP-expressing embryos were removed to clean distilled water, becoming nearly undetectable at 72 hr post-treatment (Figure 3T, 3U). These findings indicate that the GFP signals exhibited by *huORFZ* embryos most likely represent the presence or absence of stress-causing metals before morphological defects of metal-treated embryos could be observed. It should also be noted that the concentration used for chlorpyrifos, 0.03 mg/L, or 30 ppb, is in accordance with WHO drinking water guidelines. Therefore, *huORFZ* embryos could potentially be employed as a novel organismal tool to monitor chronic environmental stresses.

### Field monitoring using the transgenic line *huORFZ* embryos

**Figure 4:**
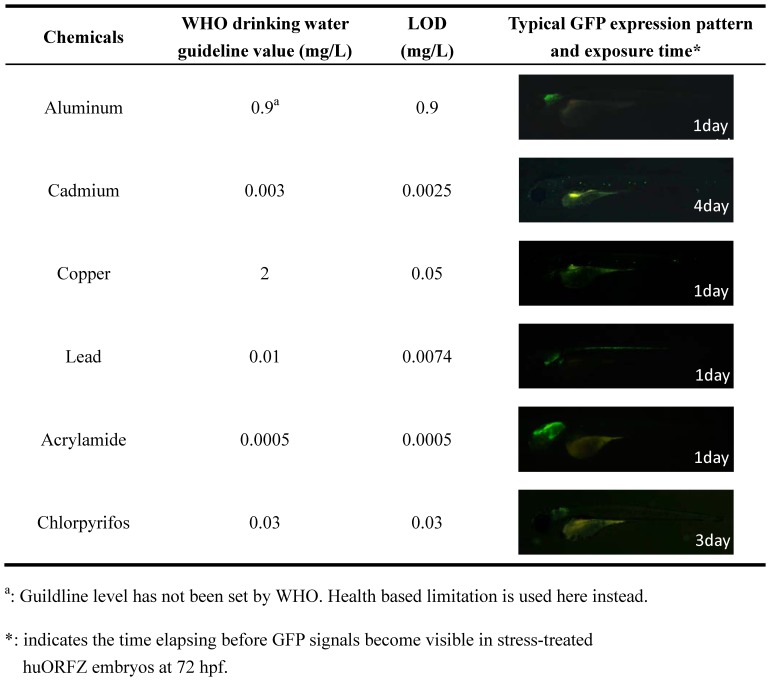
The limit of detection (LOD) of *huORFZ* embryos can reach WHO guideline values for various heavy metals and endocrine-disrupting chemicals (EDCs). LOD was defined as the lowest tested concentration that led to detectable GFP signals in more than 80% of the treated embryos after one to four days of treatment. For each kind of treatment, a representative image of a *huORFZ* embryo treated with the chemical at the LOD concentration for the period of time indicated was presented. All images are lateral views with anterior to the left.

To assess the real-world performance of *huORFZ*, we first determined the LODs for several common heavy metals and EDCs. For aluminum, copper, lead and acrylamide, we found that one-day treatments at, or below, the WHO drinking water guidelines were sufficient for the *huORFZ* to exhibit detectable tissue-specific GFP patterns. However, for cadmium and chlorpyrifos, four and three days, respectively, were required (Figure 4).

**Figure 5:**
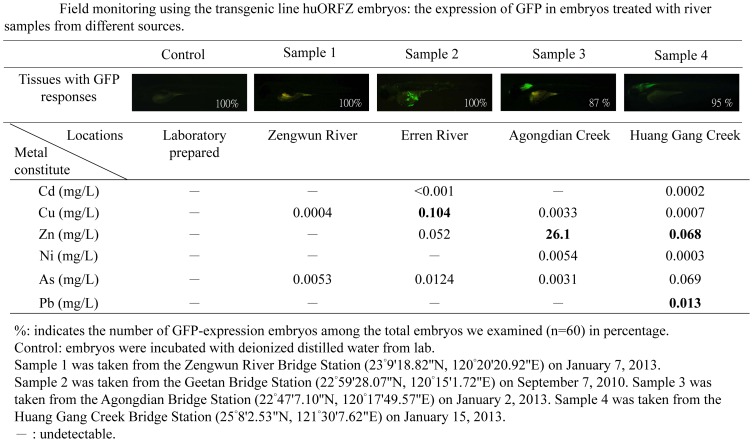
Embryos derived from transgenic line *huORFZ* provide true signals of a contaminated aquatic environment. The GFP fluorescent signal intensities induced in *huORFZ* embryos showed responses relative to different river samples collected from local waterways. In Sample 1, no GFP signal was observed in *huORFZ* embryos, consistent with WHO water safety standards. In Sample 2, the GFP response in skin tissue of *huORFZ* embryos indicated potential copper pollution. In Sample 3, GFP signals in the brain of *huORFZ* embryos corresponded to embryonic toxicity consistent with high Zn^2+^ levels. Finally, in Sample 4, the GFP response shown in CNS of *huORFZ* embryos was attributed to the presence of multiple pollutants, such as Zn^2+^ and Pb^2+^. All images are lateral views with anterior to the left.

As a proof of concept, we tested the response of *huORFZ* embryos against water samples collected from different local river basins. For comparison, the zinc, copper, cadmium, arsenic, nickel and/or lead contamination in these samples was also analyzed using conventional methods (Figures 5 and S3). Results showed the absence of GFP fluorescence in embryos incubated with water from Sample 1, indicating that the water quality in this location contains no toxicant whose levels reach beyond the WHO standard. This result was confirmed by conventional analysis. However, distinct GFP expression patterns were observed in embryos incubated separately with water from Samples 2, 3 or 4. Specifically, *huORFZ* embryos exhibited a scattered skin GFP expression pattern when treated with Sample 2, and they displayed GFP signals in brain after exposure to Sample 3. In the case of Sample 2 and Sample 3, the source of GFP expression in the *huORFZ* embryos was conclusively attributed to the respective pollutants tested. The primary source for Sample 4 was highly polluted and led to 100% mortality within 24 hr and thus required dilution. Accordingly, we diluted the sample from 4% to 5%, 10%, 20%, 40% and 80% and then used these diluted samples to treat the *huORFZ* embryos for 24 hr (data not shown). The sample diluted to 20% was then selected for use in the following study. The GFP response in the CNS of *huORFZ* embryos demonstrated a synergistic reaction to the toxicity of Zn^2+^ (0.068 mg/L) and Pb^2+^ (0.013 mg/L) in Sample 4. These results support the hypothesis that GFP expression in *huORFZ* embryos gives faithful reflections of multiple aquatic pollutants. To confirm that the expression pattern we observed in Sample 4 was indeed caused by the metal pollutants we detected, instead of unknown and untested chemicals in the water sample, we treated the *huORFZ* embryos with water containing individual ions according to their concentrations found in Sample 4. Specifically, we treated the embryos separately with As^3+^ (0.069 mg/L), Ni^2+^ (0.0003 mg/L), Pb^2+^ (0.013 mg/L) or Zn^2+^ (0.068 mg/L), as well as a mixture of all four metals. We observed that Pb^2+^alone, Zn^2+^ alone and the mixture of four metals were all capable of inducing a GFP expression pattern similar to that of Sample 4 in *huORFZ* embryos (Figure S2). These results suggested that *huORFZ* embryos are responsive to a complex sample containing mixed pollutants.

## Discussion

### The advantages and disadvantages of traditional methods

Traditionally, chemical analysis is accurate in terms of quantification of trace amount chemicals. However, it is often costly, labor-intensive and time-consuming. Such characteristics make this method ideal for producing detailed results for a limited amount of samples, but unrealistic for first-line monitoring that covers vast water bodies in large areas. Enzyme immunoassay is a relatively practical tool for on-site environmental screening [29]. However, it can only detect the targets for which detection kits are designed. The use of bioindicators, on the other hand, offers numerous advantages over the traditional methods. Most significantly, the tolerance of bioindicators for toxicants provides a biologically meaningful picture of pollution levels (Holt and Miller, 2011). To date, a growing number of identified and characterized DNA motifs that can respond to environmental stresses have emerged, including estrogen response elements (EREs) [16], [18], [30] and heat-shock protein promoters [31]–[33]. However, limited detection targets and leaked signal of the reporter gene are still problematic for these systems.

### The advantages of the *huORFZ* system

In this study, we demonstrated that the zebrafish transgenic line *huORFZ*, with the reporter gene regulated by a human *chop* uORF cassette, exhibits no detectable leakage under normal condition. The *huORFZ* system is a rapid, sensitive, and simple bioindicator able to provide a fluorescent, and, therefore, visible, signal of environmental toxicants. Since the GFP signals exhibited by *huORFZ* do not directly respond to the presence of any hazardous chemical, but rather reflect the cellular or physiological condition, *huORFZ* can be used as a first-line alarm system to detect the presence of stress-inducing chemicals, even when the chemical is not included in the standard water quality guidelines. Also, *huORFZ* embryos can be used to assess the effects of pollutants on living organisms exposed to chronic stress significantly below lethal dosages. More importantly, the expression of GFP is reversible once the exposed embryos are returned to normal physiological conditions, further supporting *huORFZ* embryos as bioindicators of stress states at the cellular level. To date, a growing number of structurally and functionally diverse groups of chemicals are being generated for industrial use. Some of these chemicals are suspected of having EDC activity, while others remain understudied. Under these circumstances, the ability of *huORFZ* to detect a broad spectrum of pollutants is an unprecedented characteristic for a transgenic animal model. Importantly, we demonstrated that one day's treatment is sufficient to identify the LODs of *huORFZ* embryos for many common pollutants, even at values lower than those specified in the drinking water guidelines recommended by WHO. For other pollutants, including Cd^2+^ and chlorpyrifos, three to four days of treatment were required for the *huORFZ* to detect WHO guideline concentration.

### The sensitivity, specificity, reproducibility and confounding factors of using huORFZ as a biomonitor

Using *huORFZ* embryos GFP signals for monitoring chemicals in water, we have to concern its sensitivity and specificity. Regarding sensitivity, in this study we have demonstrated that *huORFZ* can easily detect pollutants with near LC_10_ concentration. The LOD of *huORFZ* can even be pushed to reach WHO guideline values for various heavy metals and EDCs, even though signal strength was significantly weaker than optimal, and in several cases, the treatment time needed to be extended. Regarding specificity, the mechanism of GFP signaling in *huORFZ* is not a specific response to target toxicants. Rather, it most likely reflects the level of physiological stress in the embryo's cells or tissues. Notably, when applied as a biomonitoring system, the detection range of *huORFZ* spans multiple designated pollutants. Also, upon challenge by different pollutants, the correspondingly different GFP patterns can indicate what groups of pollutants are likely present in the water, thus improving the efficiency of subsequent chemical analysis. In this sense, we anticipate that *huORFZ* is suitable of being used as another tool supporting chemical analysis. Since many pollutants can cause similar GFP pattern in *huORFZ* embryos, the effects of mixed pollutants on *huORFZ* are not fully predictable.

Moreover, in the development of biomarker/bioindicator for pollution monitoring, we have to concern the reproducibility and the confounding factors interfering the transgenic fish's GFP responses to chemicals. Regarding reproducibility, as demonstrated in Figure S1, individual variation does exist among *huORFZ* embryos. However, as demonstrated by the percentage indicated in Figures 1A, 2A and 2B, in most the cases, more than 70% of the embryos were responsive and exhibited similar GFP patterns. We did observe that sometimes an abnormally large percentage of the embryos died under treatments with intermediate concentrations. This may have been caused by suboptimal parental health, and the results of those repetitions were discarded. Otherwise, we did not observe any significant different between repetitions. In addition, as demonstrated in Figure S2, the GFP patterns were the same in *huORFZ* exposed to Sample 4 river water as those responding to an artificial mixture containing heavy metals identical to those in the river water sample. This result also indicates that the GFP pattern of *huORFZ* is reproducible. Regarding confounding factors, the practical use of *huORZF* as a biomonitoring system could encounter a situation where certain combination of the pollutants may alter its GFP expression, leading to biased results. Also, since GFP expression likely reflects stresses at the cellular level, it is possible that certain harmful pollutants will not be detected since they do not cause direct cellular stress. To better address these concerns, we will focus our future study on understanding the mechanism and regulation of *uORF^chop^* in order to elucidate the precise physiological meaning of *huORFZ* response, as indicated by the GFP pattern.

### Physiological significance *of* the stress-specific expression pattern found in *huORFZ*

While the *chop* gene is generally regarded as an indicator of ER stress [17]–[18], the mechanism and upstream regulation of the translation inhibitory activity of *uORF^chop^* remains unclear. The fact that the GFP signals of *huORFZ* can be observed in tissues without known endogenous *ddit3* expression (skin, muscle and pronephric duct) suggests that the mechanism upstream of *uORF^chop^* may play roles beyond simply regulating *chop*. Thus, the detailed physiological meaning of the GFP signals of *huORFZ* remains to be further investigated.

### Limitations of the *huORFZ* system and future study

Currently, the utility of *huORFZ* embryos does have certain limitations. First, the reporter gene used in *huORFZ* is fluorescent-based. While the cost of fluorescent microscopy instrumentation is reasonable by the standard of a modern biology laboratory, field stations cannot be expected to bear such costs, thus potentially voiding this advantage of the *huORFZ* system. However, it is expected that different reporter genes or proteins may be used to eliminate the requirement of fluorescent microscopes. Secondly, when concentrations of the chemical treatment were reduced to near WHO drinking water guidelines, a GFP signal could still be detected, albeit at a significantly lower intensity than results otherwise obtained from treating the embryos with higher chemical concentrations. Such results are not completely unexpected since we suspect that the GFP expression of *huORFZ* reflects cellular or physiological stress, while the WHO drinking water guideline levels are generally considered relatively “safe” and are unlikely to induce significant cellular stress responses. Thirdly, the GFP patterns expressed in the arsenic-exposed *huORFZ* embryos are not always consistent. Specifically, when embryos were incubated with arsenic for 24 hr, they exhibited strong GFP signals at the lateral line system, whereas the expression patterns changed randomly when they were treated for 48 hr (data not shown). The reason for this irregularity should be the subject of further study.

## Conclusion

In this study, we demonstrated the sensitivity and versatility of the *huORFZ* system as a bioindicator for various kinds of stress-inducing pollutants in water. We also demonstrated that the *huORFZ* system performed well under real-world conditions. When fully developed, we anticipate that this *uORF^chop^*-based system can be integrated into a first-line water security system monitoring fresh water bodies and seawater against the discharge of hazardous pollutants.

## Supporting Information

Figure S1 **Images of larger field of view are used to demonstrate the general patterns and individual variability of** ***huORFZ*** **embryos treated with different heavy.** (A) huORFZ embryos were treated with embryo media, ddH2O, or DMSO for 24 and 48 hr starting at 72 hpf. (B-D) The effects of different treatment times (24 and 48 hr) with different concentrations of cadmium (0.5, 1, and 5 µM), copper (1, 2 and 3 µM) and chlorpyrifos (4.2, 4.7, and 5.83 µM) on huORFZ embryos. For chlorpyrifos treated group, at 48 hr treatments are 100% lethal. All images were taken under the Leica MZ FLIII microscope with 2x objective. All images were taken under the same exposure time, iso value and other camera settings.
(PDF)

Figure S2 **The four major metal pollutants found in the river water sample 4 are sufficient to induce** ***huORFZ*** **embryos to express the GFP signal similar to what was caused by river water sample 4.** *huORFZ* embryos were treated with water containing (A, A′) Zinc, (B, B′) Nickel, (C, C′) Arsenic, or (D, D′) Lead ion individually, or (E, E′) the water containing all four pollutants. The left panel (A, B, C, D, E) demonstrates group images taken under 2x objective while the right panel (A′, B′, C′, D′, E′) contains the images of one representative embryo of each group, taken under 4x objective. Right panel images are lateral views with anterior to the left. All scale bars are 1 mm.
(PDF)

Figure S3 **Higher resolution images of the images presented in** **Figure 5** **.** All images are exactly the same as in Figure 5, only in larger format.
(PDF)

Table S1 **Lethal concentrations for 10%, 50% and 90% mortality of 72-hpf huORFZ zebrafish embryos treated with heavy metals and endocrine-disrupting chemicals (EDCs) for 24-hr.**
(DOC)

Table S2 **River water sampling record.**
(DOC)
