# Supplementary figures and images for: Zebrafish Transgenic Line huORFZ Is an Effective Living Bioindicator for Detecting Environmental Toxicants
Source: PLoS One. 2014 Mar 3;9(3):e90160. doi: 10.1371/journal.pone.0090160 (PMC3940833; doi:10.1371/journal.pone.0090160)

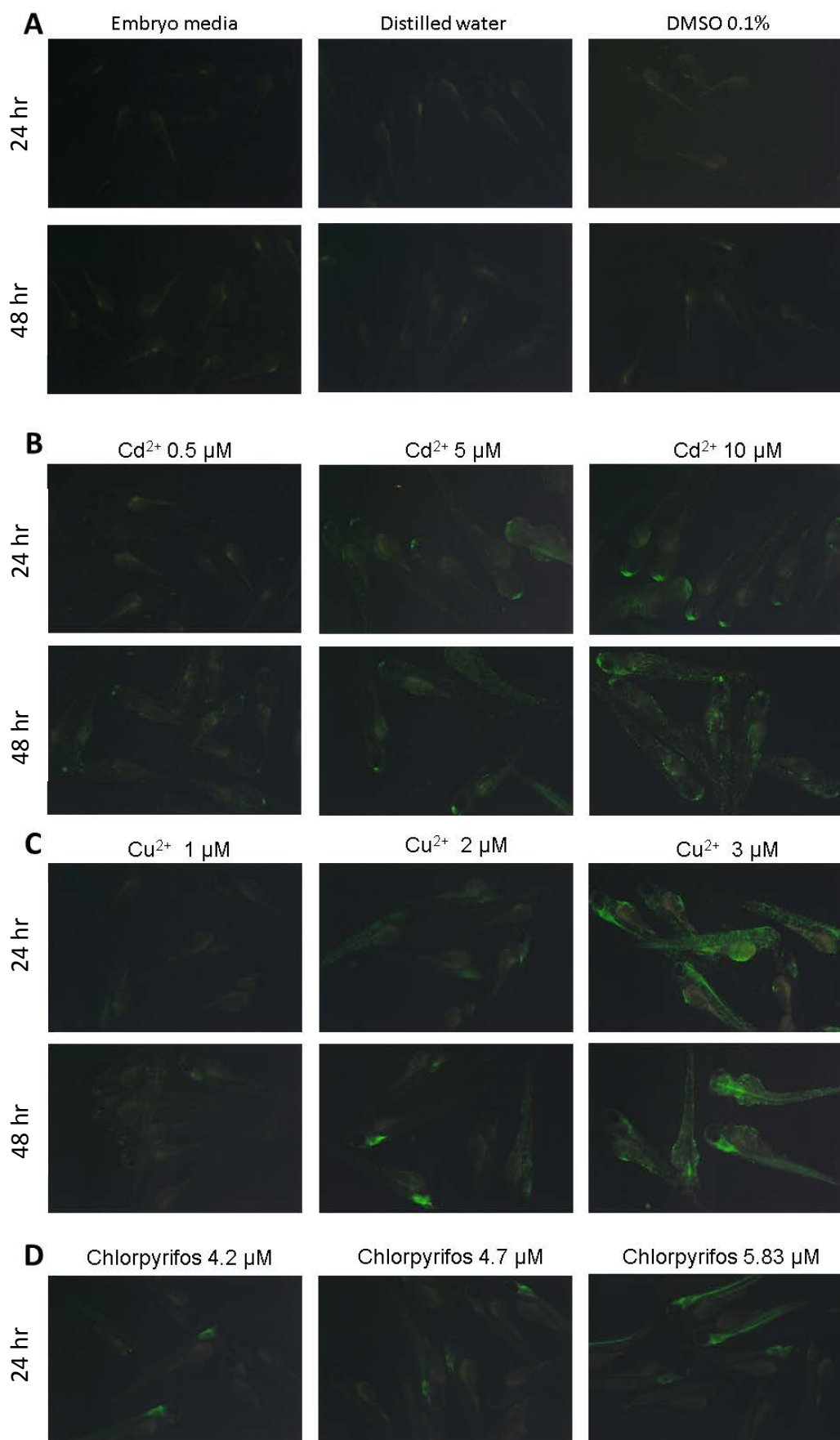

Supplement: Figure S1 — Images of larger field of view are used to demonstrate the general patterns and individual variability of huORFZ embryos treated with different heavy. (A) huORFZ embryos were treated with embryo media, ddH2O, or DMSO for 24 and 48 hr starting at 72 hpf. (B-D) The effects of different treatment times (24 and 48 hr) with different concentrations of cadmium (0.5, 1, and 5 µM), copper (1, 2 and 3 µM) and chlorpyrifos (4.2, 4.7, and 5.83 µM) on huORFZ embryos. For chlorpyrifos treated group, at 48 hr treatments are 100% lethal. All images were taken under the Leica MZ FLIII microscope with 2x objective. All images were taken under the same exposure time, iso value and other camera settings. (PDF) [file pone.0090160.s001.pdf]

$\text{Zn}^{2+}$

0.0002 mg/L

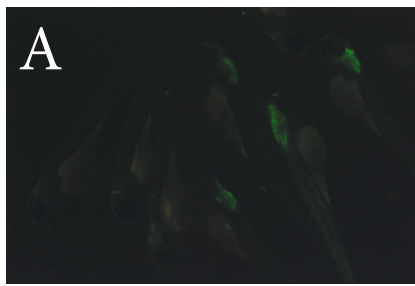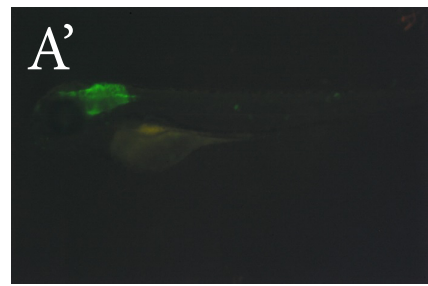

$\text{Ni}^{2+}$

0.0002 mg/L

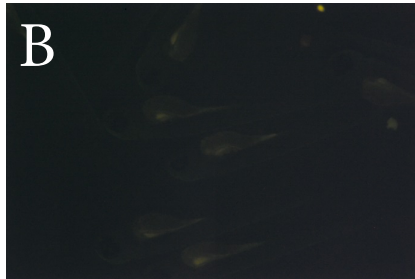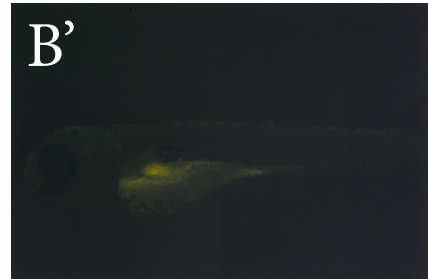

$\text{As}^{3+}$

0.0002 mg/L

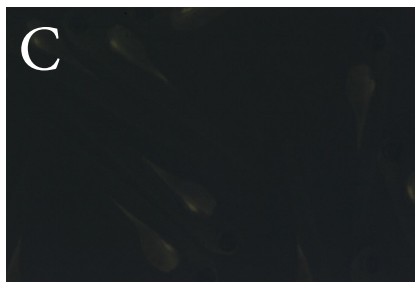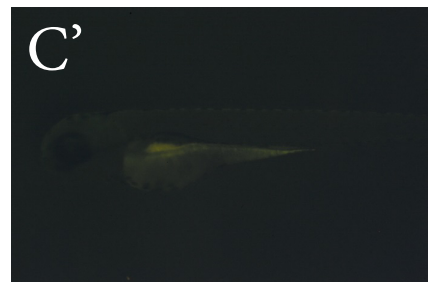

$\text{Pb}^{2+}$

0.0002 mg/L

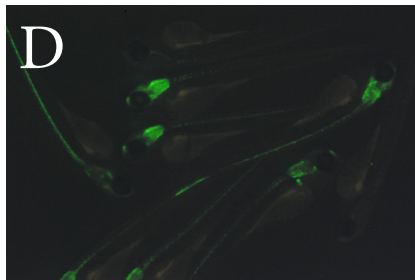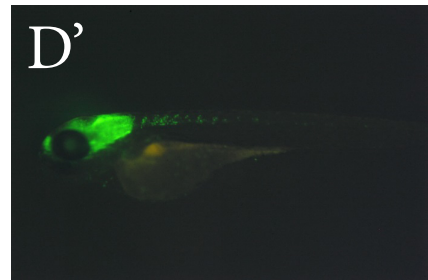

Mix

0.0002 mg/L

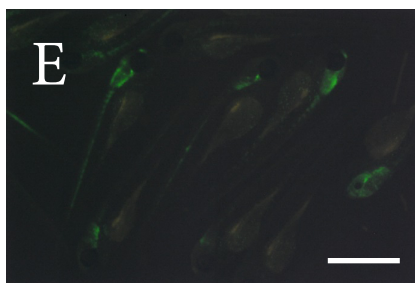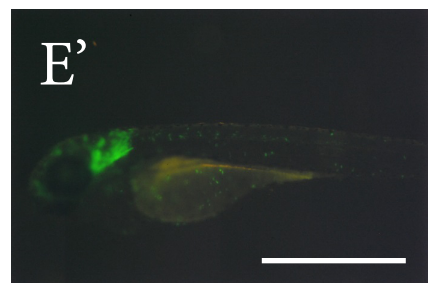

Supplement: Figure S2 — The four major metal pollutants found in the river water sample 4 are sufficient to induce huORFZ embryos to express the GFP signal similar to what was caused by river water sample 4. huORFZ embryos were treated with water containing (A, A′) Zinc, (B, B′) Nickel, (C, C′) Arsenic, or (D, D′) Lead ion individually, or (E, E′) the water containing all four pollutants. The left panel (A, B, C, D, E) demonstrates group images taken under 2x objective while the right panel (A′, B′, C′, D′, E′) contains the images of one representative embryo of each group, taken under 4x objective. Right panel images are lateral views with anterior to the left. All scale bars are 1 mm. (PDF) [file pone.0090160.s002.pdf]

Control

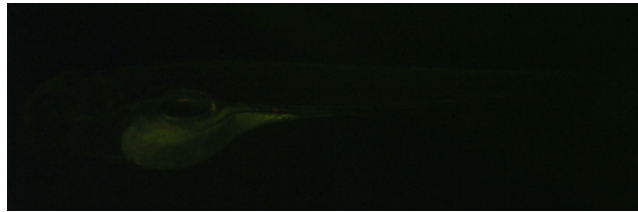

Sample 1

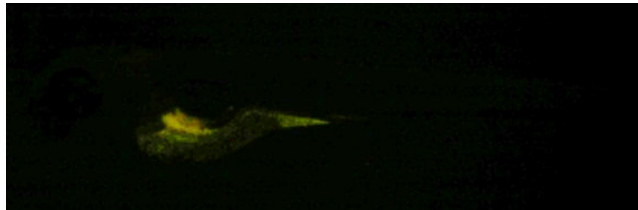

Sample 2

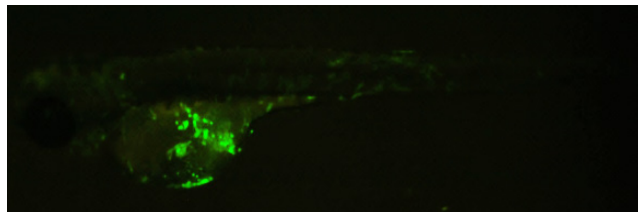

Sample 3

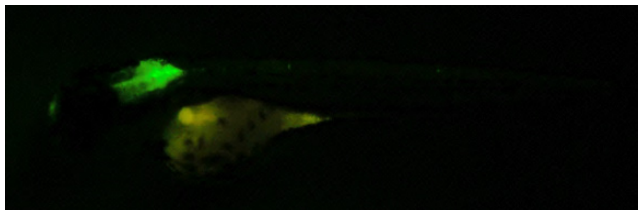

Sample 4

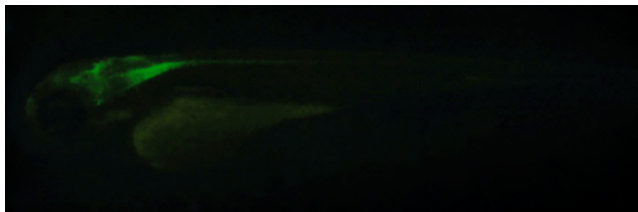

Supplement: Figure S3 — Higher resolution images of the images presented in Figure 5 . All images are exactly the same as in Figure 5, only in larger format. (PDF) [file pone.0090160.s003.pdf]
