# Supplementary material for: Zebrafish Transgenic Line huORFZ Is an Effective Living Bioindicator for Detecting Environmental Toxicants
Source: PLoS One. 2014 Mar 3;9(3):e90160. doi: 10.1371/journal.pone.0090160 (PMC3940833; doi:10.1371/journal.pone.0090160)
Supplement: Table S1 — Lethal concentrations for 10%, 50% and 90% mortality of 72-hpf huORFZ zebrafish embryos treated with heavy metals and endocrine-disrupting chemicals (EDCs) for 24-hr. (DOC) [file pone.0090160.s004.doc]

**Table S1. Lethal concentrations for 10%, 50% and 90% mortality of 72-hpf huORFZ zebrafish embryos treated with heavy metals and endocrine-disrupting chemicals (EDCs) for 24-hr**

| **Treatments** | **Selected**  **conc. (mg/L)**^a^ | **LC_10_ ( mg/L )** | **LC_50_ ( mg/L )** | **LC_90_ ( mg/L )** |
| --- | --- | --- | --- | --- |
| **heavy metals** |  |  |  |  |
| Al(III) | 2.70 | 1.35 | 6.48 | 34.53 |
| As(III) | 50 | 59.92 | 109.35 | 199.98 |
| Cd(II) | 0.56 | 0.33 | 0.58 | 1.05 |
| Co(II) | 58.93 | 34.77 | 69.50 | 137.90 |
| Cu(II) | 0.10 | 0.070 | 0.12 | 0.21 |
| Li(I) | 242.9 | 229.02 | 260.25 | 347 |
| Ni(II) | 58.69 | 34.63 | 69.84 | 138.51 |
| Pb(II) | 0.76 | 0.41 | 1.04 | 24.86 |
| Zn(II) | 22.24 | 7.20 | 22.24 | 67.36 |

| **EDCs** |  |  |  |  |
| --- | --- | --- | --- | --- |
| Acrylamide | 26.98 | 22.01 | 27.69 | 35.50 |
| Atrazine | 32.35 | 36.67 | 40.98 | 49.61 |
| Carbofuran | 1.34 | 0.83 | 1.34 | 2.23 |
| Chlorpyrifos | 1.65 | 1.40 | 1.75 | 2.21 |
| Dimethoate | 1375 | 1263 | 1420 | 1595 |
| Glyphosate | 18.60 | 21.97 | 27.05 | 33.81 |
| Methoxychlor | 4.87 | 3.56 | 4.87 | 6.50 |

^a^ Concentration used in this study ( see Figure 1) is below or equal to the LC_50_ value.
LC values of each treatment were estimated using PriProbit.
