# Supplementary material for: Zebrafish Transgenic Line huORFZ Is an Effective Living Bioindicator for Detecting Environmental Toxicants
Source: PLoS One. 2014 Mar 3;9(3):e90160. doi: 10.1371/journal.pone.0090160 (PMC3940833; doi:10.1371/journal.pone.0090160)
Supplement: Table S2 — River water sampling record. (DOC) [file pone.0090160.s005.doc]

**Table S2. River water sampling record**

| **Sample Number** | **Station Name** | **Station Coordinate** | **Sampling Date** | **Sampling Personnel** |
| --- | --- | --- | --- | --- |
| 1 | Zengwun River Bridge Station | 23°9'18.82''N, 120°20'20.92''E | January 7, 2013 | CENPRO |
| 2 | Geetan Bridge Station | 22°59'28.07''N, 120°15'1.72''E | September 7, 2010 | CENPRO |
| 3 | Agongdian Bridge Station | 22°47'7.10''N, 120°17'49.57''E | January 2, 2013 | CENPRO |
| 4 | Huang Gang Creek Bridge Station | 25°8'2.53"N, 121°30'7.62"E | January 15, 2013 | H.L. Huang |
